# Repurposing metabolic regulators: antidiabetic drugs as anticancer agents

**DOI:** 10.1186/s43556-024-00204-z

**Published:** 2024-09-28

**Authors:** Yogita Dhas, Nupur Biswas, Divyalakshmi M.R., Lawrence D. Jones, Shashaanka Ashili

**Affiliations:** 1 Rhenix Lifesciences, Hyderabad, 500038 Telangana India; 2 CureScience, 5820 Oberlin Dr, Suite 202, San Diego, CA 92121 USA

**Keywords:** Diabetes mellitus, Cancer, Drug repurposing, Antidiabetic drugs

## Abstract

Drug repurposing in cancer taps into the capabilities of existing drugs, initially designed for other ailments, as potential cancer treatments. It offers several advantages over traditional drug discovery, including reduced costs, reduced development timelines, and a lower risk of adverse effects. However, not all drug classes align seamlessly with a patient's condition or long-term usage. Hence, repurposing of chronically used drugs presents a more attractive option. On the other hand, metabolic reprogramming being an important hallmark of cancer paves the metabolic regulators as possible cancer therapeutics. This review emphasizes the importance and offers current insights into the repurposing of antidiabetic drugs, including metformin, sulfonylureas, sodium-glucose cotransporter 2 (SGLT2) inhibitors, dipeptidyl peptidase 4 (DPP-4) inhibitors, glucagon-like peptide-1 receptor agonists (GLP-1RAs), thiazolidinediones (TZD), and α-glucosidase inhibitors, against various types of cancers. Antidiabetic drugs, regulating metabolic pathways have gained considerable attention in cancer research. The literature reveals a complex relationship between antidiabetic drugs and cancer risk. Among the antidiabetic drugs, metformin may possess anti-cancer properties, potentially reducing cancer cell proliferation, inducing apoptosis, and enhancing cancer cell sensitivity to chemotherapy. However, other antidiabetic drugs have revealed heterogeneous responses. Sulfonylureas and TZDs have not demonstrated consistent anti-cancer activity, while SGLT2 inhibitors and DPP-4 inhibitors have shown some potential benefits. GLP-1RAs have raised concerns due to possible associations with an increased risk of certain cancers. This review highlights that further research is warranted to elucidate the mechanisms underlying the potential anti-cancer effects of these drugs and to establish their efficacy and safety in clinical settings.

## Introduction

The evolving relationship between metabolic disorders and oncogenesis signifies a paradigm shift extending beyond traditional disease management. Diabetes and cancer, both prevalent and impactful, are on the rise globally [1–3]. Diabetes, characterized by elevated blood sugar levels, affects a substantial global population, with approximately 536.6 million individuals in 2021, projected to rise to 783.2 million by 2045 [4]. Concurrently, cancer stands as a primary contributor to global mortality, with GLOBOCAN 2022 estimating 20 million new cases and 9.7 million deaths in 2022 [5]. This dual escalation of diabetes and cancer underlines the pressing need for comprehensive research and effective strategies to address the growing burden of diabetes and cancer worldwide. The interplay between diabetes and cancer is a complex and multifaceted relationship drawing increased attention in oncology and endocrinology. It involves intricate associations at molecular, metabolic, and epidemiological levels [2, 3]. It is essential to acknowledge this dynamic interplay because shared risk factors and biological processes mutually shape and influence their interconnected growth. The coexistence of diabetes and cancer often stems from common risk factors like obesity, sedentary lifestyle, and aging, fostering a chronic inflammatory state and insulin resistance, thereby creating a favorable environment for both conditions [1–3, 6]. In fact, the correlations between diabetes, cancer along with cardiovascular diseases are also reported [7].

The intricate interplay between diabetes and cancer involves shared molecular pathways like insulin signaling, AMP-activated protein kinase (AMPK), and the mammalian target of rapamycin (mTOR) [8–10]. Epidemiological studies consistently associate diabetes with an increased risk of specific cancers, including pancreas, colon, rectum, liver, breast, bladder, endometrium (uterine), and prostate cancers [11]. This intersection has become a focal point in recent research, leading to the exploration of repurposing antidiabetic drugs for cancer therapy. Drug repurposing involves identifying new therapeutic uses for existing drugs initially approved for different indications. This approach accelerates drug discovery by leveraging drugs with established safety profiles and human approvals, reducing time and costs. However, barriers, including safety concerns, intellectual property issues, and regulatory hurdles, need addressing [9, 12–16]. Despite challenges, the potential benefits drive research, offering a pragmatic solution for unmet medical needs. Repurposing antidiabetic drugs in cancer therapy represents a paradigm shift, moving beyond glucose control to actively impede tumor growth. The multifaceted mechanisms position these drugs as appealing candidates for combination therapies, necessitating thorough exploration to realize their potential in oncology [8–10].

Metabolic regulation refers to the process through which metabolic pathways are regulated for the purpose of maintaining glucose homeostasis [17]. Metabolic regulators, often referred as, antidiabetic drugs, originally designed for glucose regulation emerge as potent modulators of key cellular pathways in cancer [8, 10]. Cancer cells, characterized by the Warburg effect and altered metabolism, face growth inhibition from drugs like metformin, disrupting their energy balance [18, 19]. Beyond glucose control, antidiabetic medications exhibit anti-inflammatory properties, offering the potential to reshape the cancer microenvironment and enhance the antitumor immune response [8, 10, 19]. Certain drugs, like TZDs, demonstrate antiangiogenic effects, hinting at their potential to impede tumor vascularization and restrain growth [8, 19]. Thus, repurposing these antidiabetic drugs for cancer treatment holds promising prospects. Combining antidiabetic drugs with conventional cancer therapies shows promise in preclinical and clinical studies, with observed synergistic effects enhancing treatment effectiveness while mitigating side effects [3, 20–22]. Observational studies and clinical trials offer valuable insights, but meta-analyses and systematic reviews are essential for a comprehensive overview, emphasizing the need for further research to establish clear guidelines for the application of antidiabetic drugs across various cancer types [8, 10, 19].

In light of this shared landscape, this review delves into repurposing synthetic pharmaceutical antidiabetic drugs in cancer therapy, shifting the focus from glucose control to growth inhibition. By exploring shared molecular mechanisms and signaling pathways between diabetes and cancer, the aim is to provide insights into the dual benefits of these drugs. We have looked into the repurposed use of antidiabetic drugs across different diseases and then focused on cancer. The drugs under consideration include metformin, sulfonylureas, sodium-glucose cotransporter 2 (SGLT2) inhibitors, dipeptidyl peptidase 4 (DPP-4) inhibitors, glucagon-like peptide-1 receptor agonists (GLP-1RAs), thiazolidinediones (TZDs), and α-glucosidase inhibitors. Also, this review is focused to type 2 diabetes mellitus (T2DM) as almost 90% of occurrence of diabetes belong to T2DM type [23].

## Linkage between cancer and diabetes

As diabetes and cancer share multiple common risk factors like obesity, physical inactivity, smoking, alcohol consumption, etc. [24] antidiabetic drugs are considered as first-choice as repurposed drug of cancer. The outcomes shared between these two diseases include hyperinsulinemia, hyperglycemia and inflammation (Table 1) [25]. Also, there exists several molecular pathways which are affected by both diabetes and cancer [26].

**Table 1:** Causal factors in diabetes contributing to cancer development

Triggers	Mediators	Effectors
Chronic inflammation	Inflammatory cytokines, such as TNF-α and IL-6	Increased cell proliferation, angiogenesis, and invasion
Insulin resistance	Insulin signaling pathways	Increased cell proliferation and survival
Hyperglycemia	Advanced glycation end products (AGEs)	DNA damage, oxidative stress, and inflammation
Obesity	Adipokines, such as leptin and adiponectin	Increased cell proliferation, inflammation, and angiogenesis

There are multiple signaling as well as metabolic pathways which modulate fundamental cellular mechanisms and are shared by diabetes and cancer. It paves way the possibility of using antidiabetic drugs in cancer. The major pathways are Wnt (wingless-type MMTV integration site family) signaling pathways, mammalian target of rapamycin (mTOR) pathway, transforming growth factor (TGF)—β signaling, interleukin (IL)-6 signaling, hypoxia inducible factor (HIF) signaling and platelet-derived growth factor (PDGF) signaling [3, 6].

Wnt signaling pathways include canonical Wnt/β-catenin pathway and non-canonical β-catenin independent pathways. These pathways are involved in cell proliferation, migration and organogenesis during embryonic development [27]. Increased glucose level is known to affect Wnt signaling pathway and hence modulates cell proliferation [28]. mTOR signaling pathway integrates extracellular and intracellular signals and regulates various cellular mechanisms like metabolism, cell growth and proliferation [29]. TGF-β signaling pathway also regulates various cellular functions including glucose tolerance, energy homeostasis and can promote tumorigenesis and metastasis. IL-6 cytokine is also involved both in diabetes and cancer by controlling glucose homeostasis, insulin sensitivity as well as tumorigenesis in different cancers [30]. Through HIF signaling pathway, HIF proteins regulate oxygen homeostasis. In diabetes, as tissues are hypoxic, insufficient activation of HIF signaling leads to different complications [31]. In the case of cancer, HIF can promote tumorigenesis by adapting tumor cells to hypoxia [32]. Mitogen-activated protein kinase (MAPK) pathway regulating metabolic homeostasis is also involved both in diabetes and cancer. Apart from the signaling and mitogenic pathways, metabolic pathways like glycolysis, glutaminolysis play crucial role in diabetes as well as cancer [33, 34].

## Repurposed drugs in cancer

**Fig. 1:**
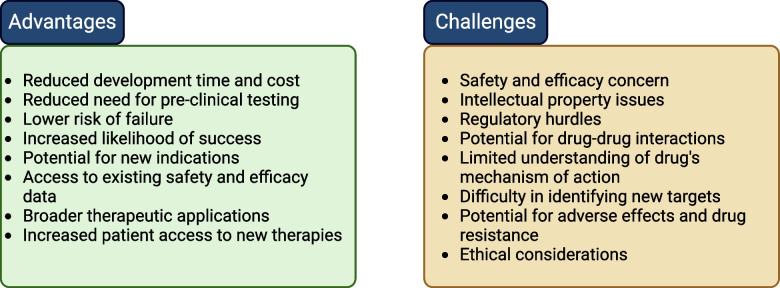
Advantages and challenges in drug repurposing

As cancer possess a crucial challenge in healthcare repurposed use of different types of drugs have been explored to combat cancer and is also documented [8, 13, 35, 36]. Figure 1 illustrates the advantages and challenges associated with drug repurposing. Broadly, the hallmarks of cancer are targeted for repurposed use of drugs [36]. Cancer originates due to the failure of immune system to combat mutated proteins. Autoimmune diseases and cancer are well related. Some autoimmune diseases show increased risk of cancer [37, 38]. Hence, using immunotherapy for cancer patients having auto-immune disease is a challenge. Apart from antidiabetic drugs, various other drugs have been repurposed to treat cancer. Statins, commonly used for lowering blood cholesterol, are being used in clinical trials for treating some cancer as it elevates P53 protein [36, 39]. Benjamin et al. reviewed that cardiovascular drugs including aspirin, statins do not have benefit in treating cancer [40]. Among the anti-inflammatory drugs, Non-steroid Anti-inflammatory Drugs (NSAIDs) are also repurposed for cancer. NSAIDs like celecoxib, ibuprofen, cyclovalone, diclofenac are known to exhibit anti-tumor properties and used for clinical trials [41]. Cancer patients being infectious-prone are treated different types of antibiotics which often helps in treating cancer. Several antibiotics and antiviral drugs also studied in clinical trials for treating cancer, however the outcomes are diverging [42]. Proton pump inhibitors which are usually used to treat acid-related disorders are often associated with increased risk of cancer [43]. On the contrary, Proton pump inhibitors improved survival rates for breast cancer patients [44]. So there exists contradictory reports on the repurposed use of different drugs and it requires more pre-clinical and clinical studies [36]. Because of the broad landscape of shared molecular pathways, antidiabetic drugs are considered as potential candidate of repurposed drugs for cancer.

## Repurposed use of antidiabetic drugs

Apart from cancer, diabetes patients suffer from many other comorbidities like obesity, dyslipidemia, hypertension, neurological disorders, cardiovascular diseases, and renal diseases. Dysregulation of metabolic pathways disturbs glucose homeostasis which results in multiple disease conditions. Hence metabolic regulators, referred as, antidiabetic drugs have been repurposed used for different disease scenarios specially for the diseases which frequently co-occur with diabetes [45]. These studies are also extended to the non-diabetic patients. Among the co-morbidities, the cognitive impairment and neurodegenerative disorders are linked with brain insulin resistance [46, 47]. Preclinical and clinical studies are conducted for metformin, GLP-1RAs, SGLT2 inhibitors, and others. These studies highlight the challenge of overcoming blood–brain barrier [47]. In the case of Parkinson’s disease (PD), GLP-1RAs have shown to reduce severity in a trial of 60 patients over 48 weeks [48]. Another neurodegenerative disease, Alzheimer’s disease (AD) also shares molecular mechanisms with T2DM. Metformin, TZDs have shown some positive effects in treatment of AD [49–51]. GLP-1RAs, SGLT2 inhibitors, DPP4 inhibitors, and metformin have also been studied for their cardioprotective roles in both diabetic and non-diabetic cardiovascular patients. The trials indicate the beneficial roles of GLP-1RAs and SGLT2 inhibitors along with confirmed safety. However, the underlying molecular mechanisms is still not well understood [52]. Diabetes patients often develop renal diseases like diabetic nephropathy. Roles of SGLT2 inhibitors and GLP1-RAs are promising in this context. SGLT2 inhibitors reduces risk of lower glomerular filtration rate (GFR). GLP-1RAs reduces risk of macroalbuminuria [53]. Effects of SGLT2 inhibitors, GLP-1RAs and DPP4 inhibitors were explored in the case of diabetes retinopathy but the conclusion could not be drawn [54, 55]. Antidiabetic drugs have also been explored in the case of auto-immune diseases like rheumatoid arthritis (RA). TZDs have demonstrated protective effects on incidence of RA as genetic variant of TZDs target gene is associated with the mechanism [56]. A population based study also reports use of TZDs reduces risk of RA in T2DM patients [57]. Metformin was explored for the treatment of inflammatory bowel disease and preclinical studies were promising. However, clinical trials reported conflicting results [58, 59]. Metformin has also shown its effectiveness by increasing pregnancy probability for women suffering from polycystic ovary syndrome (PCOS) [60]. Metformin also lowers the rate of ovarian hyperstimulation syndrome (OHSS) but short-term use may not be effective [61–63]. Among all antidiabetic drugs, metformin is the definitely most repurposed used. It is considered as a potential anti-aging drug and has been studied for its role to extend healthspan and lifespan [64, 65]. Pre-clinical and clinical studies show metformin induces anti-aging benefits by regulating cellular metabolism [66]. As metformin provides a protection against cognitive decline, it helps to defer aging induced cognitive decline [67]. Antidiabetic drugs have also been used against pathogens. DPP4-inhibitor, sitagliptin has shown potential target specific and safe inhibitor of bacterial virulence specifically of *Serratia marcescens* [68]. Different types of antidiabetic drugs conjugated with nanoparticles have been used as antimicrobial agents [69].

## Repurposed use of antidiabetic drugs for cancer

**Fig. 2:**
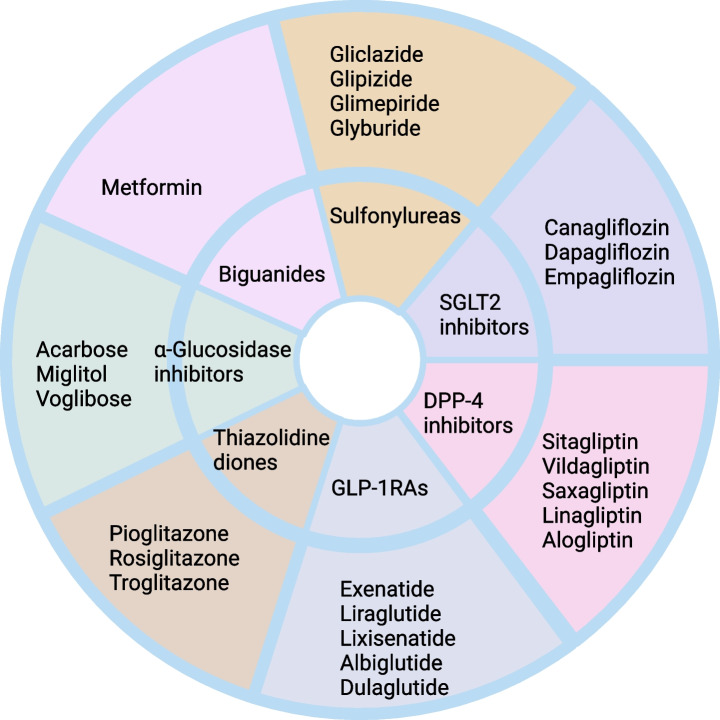
Different classes of antidiabetic drugs (inner circle) and names of drugs (outer circle) which are repurposed used to treat cancer

Among different types of antidiabetic drugs, biguanides, specifically metformin is the most frequently prescribed first-line drug of T2DM [70]. The second line drugs including sulfonylureas, SGLT2 inhibitors, DPP-4 inhibitors, GLP-1RAs, thiazolidinedione (TZD), and α-glucosidase inhibitors are also used for lowering glucose [71–73]. In this review, we have focused on these major oral medications. Biguanides primarily reduces glucose production in liver [74]. Metformin accumulates at the mitochondria of liver cell and suppresses ATP production by inhibiting mitochondrial respiratory chain complex I. Lack of ATP suppresses gluconeogenesis [75, 76] On the other hand, sulfonylureas stimulate insulin production in pancreas. To increase insulin release, sulfonylureas close ATP sensitive K-channels in pancreatic beta-cell membrane. It also plays a role to lower hepatic clearance of insulin [77, 78]. SGLT2 inhibitors also reduce blood glucose by reducing renal tubular glucose reabsorption [79]. Glucose-dependent insulinotropic polypeptide (GIP) and glucagon-like peptide-1 (GLP-1) are two major incretin hormones which stimulate insulin secretion and is referred as incretin effect. In T2DM, incretin effect is disturbed [80]. In T2DM, the use of GLP-1RAs enhance action of GLP-1. In another approach, DPP-4 inhibitors are also used to enhance levels of active GLP-1 [81]. On the other hand, TZDs insulin sensitizers and modulate insulin action and sensitivity in tissues [82]. α-glucosidase inhibitors prevent digestion of complex carbohydrates at small intestine [83]. So we observe, there exists wide variation in the mechanism of action of different anti-diabetic drugs. Hence, their repurposed use in cancer is also challenging leading to diverging outcomes as discussed in the following sections of this review. Figure 2 illustrates the major antidiabetic drug classes and drugs repurposed for cancer.

### Metformin

**Fig. 3:**
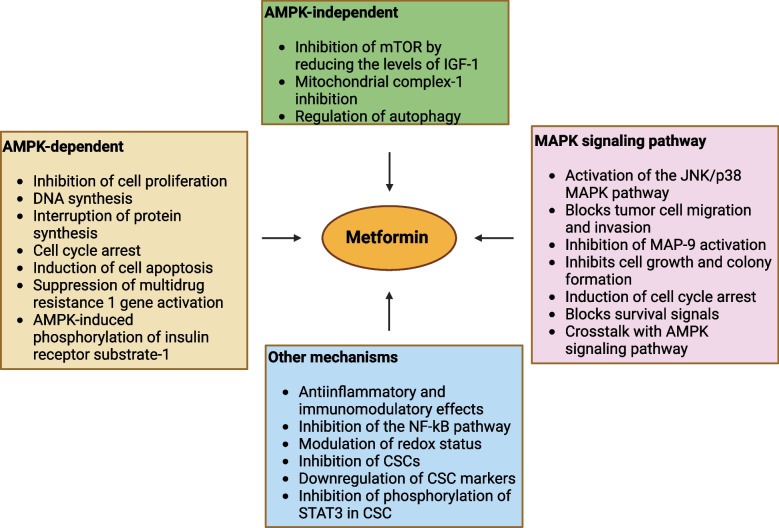
Potential anticancer effects of metformin and pathways targeting tumor growth. AMPK, AMP-activated protein kinase; mTOR, mammalian target of rapamycin; IGF-1, Insulin-like growth factor 1; MAPK, Mitogen-activated protein kinase; MMP 9, Matrix metalloproteinase-9; NF-κB, Nuclear factor kappa B; CSCs, Cancer stem cells; STAT3, Signal transducer and activator of transcription 3

Metformin, a biguanide derivative primarily used to lower blood glucose in T2DM, acts by reducing hepatic gluconeogenesis, improving insulin sensitivity, and enhancing peripheral glucose uptake [76]. The concept of repurposing metformin as an anti-cancer drug has gained traction in the last 15 years. Observational studies suggest that metformin use is linked to reduced cancer risk and decreased cancer-related and all-cause mortality [84, 85]. Numerous meta-analyses support its efficacy in lowering cancer incidence and improving outcomes, either as a single agent or in combination [8, 86, 87]. Metformin demonstrates significant efficacy in reducing the incidence and improving survival rates across various cancers, including pancreatic, colorectal, gastric, liver, breast, endometrial, lung, and prostate cancers [8, 20, 35, 86, 88]. Its anti-cancer effects are attributed to targeting multiple cellular pathways, including AMP-activated protein kinase (AMPK), AKT/mTOR signaling, and fatty acid synthesis. Metformin activates AMPK, a key regulator of cellular energy metabolism, and inhibits the AKT/mTOR pathway, resulting in reduced cancer cell growth and proliferation [8, 19, 20, 86, 87] (Fig. 3, Table 2). Additionally, metformin-induced AMPK activation decreases cancer cell proliferation through mechanisms involving activation of cMYC, Hypoxia-Inducible Factor (HIF)-1, and DICER1 [89]. Notably, metformin inhibits mTOR activation via Rag GTPases independently of AMPK and TSC1/2 [90]. In lung cancer cells, metformin induces apoptosis by JNK/p38 MAPK pathway and upregulating GADD153 gene [91]. Metformin also modulates tumor-infiltrating immune cells within tumor microenvironment (TME). It can exhibit both tumor-suppressive and tumor growth promoting roles [92]. The tumor-suppressive role includes affecting macrophage polarization and proliferation of immune cells CD8 + T cells and natural killer cells promote tumor growth [92–94].

**Table 2:** Comprehensive review of antidiabetic drugs in cancer therapy

Medication	Mechanism of action in diabetes	Potential impact on cancer risk	Evidence	Clinical trials	Cancer types studied	Limitations of the current evidence
*Biguanides* Metformin	Decreases hepatic glucose production and increases glucose uptake by muscle and adipose tissue by activating AMPK [76]	Decreases cancer cell proliferation and survival by inhibiting the AMPK/mTOR pathway; Reduces glucose, IGF-1, insulin, and various inflammatory factors [95]	Retrospective studies and animal models suggest a protective effect against cancer [96]	Numerous ongoing trials evaluated metformin as a potential for cancer therapy [97]	Breast, colorectal, endometrial, prostate, ovarian, lung, liver, kidney, oral, gastric, pancreatic, bladder, thyroid, cervical, esophageal cancer, HNSCC, brain tumor [98]	Limited data from human studies; potential confounding factors in retrospective studies; need more RCTs. Some observational studies affected by time-related biases exaggerated the benefits of metformin [99]
*Sulfonylureas* Gliclazide Glipizide Glimepiride Glyburide	Stimulate insulin secretion from pancreatic beta cells by closing ATP-sensitive potassium channels [100]	Sulfonylureas are insulin secretagogues and increased levels of IGF and insulin appear to stimulate the development of tumors [101]	Some retrospective studies suggest an association with increased cancer risk [102]	Six clinical trials evaluating sulfonylureas and its association with cancer risk [103]	Pancreatic, breast, bladder cancer	Limited data from human studies; and conflicting data from retrospective studies; need more research to elucidate the potential link between sulfonylureas and cancer risk [104]
*SGLT2 inhibitors* Canagliflozin Dapagliflozin Empagliflozin	Inhibit SGLT2, a protein that reabsorbs glucose from the urine [105]	Decrease tumor invasion and metastasis, and inhibit tumor development and proliferation [106]	Preclinical data hints at the potential benefits of combining SGLT2 inhibitors with standard chemotherapy [107]	25 clinical trials evaluating SGLT2 inhibitors for cancer therapy [108]	Breast, endometrial, colorectal, bladder, lung, pancreatic, head and neck cancer, brain tumor	Limited data from human studies; need more clinical trials to evaluate the efficacy of SGLT2 inhibitors in cancer treatment [109]
*DPP-4 inhibitors* Sitagliptin Vildagliptin Saxagliptin Linagliptin Alogliptin	Inhibit DPP-4, an enzyme that breaks down incretins, hormones that stimulate insulin secretion [110]	May decrease cancer risk by inhibiting inflammation and oxidative stress; exhibited potential to inhibit tumor growth in cancer patients [111]	Some preclinical data suggests potential anti-cancer effects and improvement in survival rates [112]	25 clinical trials evaluating DPP-4 inhibitors for cancer therapy [113]	Pancreatic, liver cancer, leukemia	Limited data from human studies need more clinical trials to evaluate the efficacy of DPP-4 inhibitors in cancer treatment [114]
*GLP-1RAs* Exenatide Liraglutide Lixisenatide Albiglutide Dulaglutide	Bind to the GLP-1 receptor, stimulating insulin secretion and inhibiting glucagon secretion [115]	May decrease cancer risk by inhibiting cell proliferation and inducing apoptosis [116]	Some preclinical data suggests a reduction in the overall risk of pancreatic cancer, but clinical data is lacking [117]	No ongoing trials specifically evaluating GLP-1RAs for cancer therapy [113]	Pancreatic cancer	Limited data from human studies; need more clinical trials to evaluate the efficacy of GLP-1RAs in various types of cancer [118, 119]
*Thiazolidinediones* Pioglitazone Rosiglitazone Troglitazone	Improve insulin sensitivity by increasing insulin receptor activity [120]	May decrease cancer risk by reducing inflammation and oxidative stress [121]	Some preclinical data suggests potential anti-cancer effects, but limited clinical evidence is available [122]	No ongoing trials specifically evaluating TZDs for cancer therapy [113]	Bladder, lung, liver, urothelial, gastric, esophageal cancer, melanoma, HNSCC	Limited data from human studies; lack of consistent data regarding the antiproliferative effects of TZDs across various in vitro and clinical studies [123]
*α-Glucosidase inhibitors* Acarbose Miglitol Voglibose	Inhibit α-glucosidase, an enzyme that breaks down carbohydrates in the digestive tract; reduction of postprandial hyperglycemia by suppressing glucose absorption [124]	Delays the absorption of carbohydrates in the intestines and reduces glucose availability to cancer cells, which may decrease cancer risk [125]	Some preclinical data reported a reduction in cancer risk, but clinical evidence is lacking [126]	No ongoing trials specifically evaluating α-glucosidase inhibitors for cancer therapy [113]	Pancreatic cancer	Limited data from human studies; need for more clinical trials to evaluate the efficacy of α-glucosidase inhibitors in cancer treatment [127]

*AMPK* 5ʹ AMP-activated protein kinase, *DPP-4* dipeptidyl peptidase 4, *GLP-1* glucagon-like peptide 1, *GLP-1RA* GLP-1 receptor agonist, *RCT* randomized controlled trial, *SGLT2* Sodium-glucose cotransporter 2, *HNSCC* head and neck squamous cell carcinoma, *IGF-1* insulin-like growth factor 1

Moreover, metformin hampers the Warburg effect, known to be exhibited by tumors, where tumor cells produce ATP by glycolysis rather than oxidative phosphorylation [19, 128]. According to in-vivo investigations on hepatocellular carcinoma xenografts, metformin enhances cellular oxygenation capacity, reduces mitochondrial oxygen consumption, and inhibits hypoxia-induced HIF-1α [129]. It also curtails insulin-driven tumorigenesis via the IGF-1 signaling pathway and boosts anti-tumor T-cell activity while suppressing immunosuppressive cells, potentially enhancing immunotherapies [19, 87] (Fig. 3). Preliminary findings from clinical trials suggest positive impacts on tumor growth markers, though the effect on survival rates remains uncertain [130–132]. However, studies on breast cancer patients using metformin as adjuvant drug found no benefit [133]. It has been reported that the combined therapy of metformin and PD-1 blockade for melanoma exhibits a tumor size threshold. Additionally, improved outcomes were seen among patients with early-stage non-small cell lung cancer (NSCLC) or those who took metformin before their NSCLC diagnosis. Therefore, this suggests that metformin should be considered for use as soon as cancer is diagnosed, or even in individuals at high risk of developing cancer, if feasible [19]. A French prospective multicentric randomized Phase II study of docetaxel plus metformin in metastatic castration-resistant prostate cancer observed that the addition of metformin failed to improve the standard docetaxel regimen in treating this condition [134]. Thus, the effects of metformin have also wrongly been assessed in several trials and hence observational studies with rigorous approaches are needed [99]. Considering tumor histology and the stage of cancer is crucial when contemplating metformin therapy.

### Sulfonylureas

Sulfonylureas, a class of oral hypoglycemic agents used for treating T2D, stimulate insulin secretion from pancreatic β-cells [78]. Recently, the potential repurposing of sulfonylureas for cancer treatment has gained attention. However, the association between sulfonylureas and cancer risk is complex [10, 101, 135]. Some studies suggest an elevated cancer risk in T2DM patients using sulfonylureas, possibly influenced by metformin use as a comparator [135–137]. In contrast, Haggstrom et al., 2017 reported decreased cancer risk in male T2DM patients using sulfonylureas compared to those not receiving antidiabetic drugs [138]. These contradictory findings may stem from divergent systemic or off-target effects of various sulfonylureas. Pathways triggered by endogenous hyperinsulinemia in diabetes may accelerate cancer development. Therapies like sulfonylureas, which can induce hyperinsulinemia, are theorized to increase the risk of cancer. Sulfonylureas, as insulin secretagogues, may elevate IGF and insulin levels, promoting tumor development [139]. Retrospective analyses of the relationship between sulfonylurea drugs and cancer risk and mortality have yielded conflicting or inconclusive results [137, 140, 141].

**Fig. 4:**
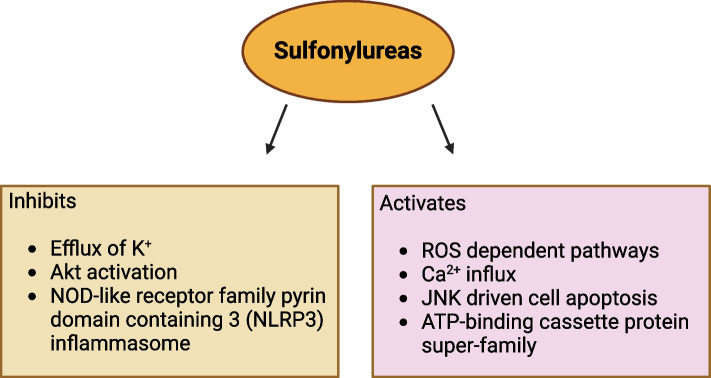
Potential anticancer effects of sulfonylureas and targeted pathways. ROS, Reactive oxygen species; Akt, Ak strain transforming; JNK, c-Jun N-terminal kinases; NOD, Nucleotide binding and oligomerisation domain

Glyburide, a frequently prescribed sulfonylurea for T2DM treatment, has been shown to potentially exhibit anti-cancer properties. This is achieved through the activation of reactive oxygen species (ROS)-dependent pathways, leading to c-Jun N-terminal kinase (JNK)-driven cell apoptosis (Fig. 4). Additionally, glyburide inhibits Akt activation, as demonstrated by Qian and colleagues in 2008. Moreover, glyburide could potentially enhance the anti-cancer effect by modulating ATP-binding cassette protein super-family and ATP-sensitive potassium channels [142]. Recent studies using non-diabetic mice models also suggested that glyburide might inhibit the NOD-like receptor family pyrin domain containing 3 (NLRP3) inflammasome, leading to a reduction in inflammation-related lung tumor development [143] (Table 2).

In a retrospective observational study involving 1277 participants, T2DM patients treated with gliclazide exhibited a lower risk of cancer mortality compared to those treated with glibenclamide [102]. Another study by Lee et al. reported a significantly higher risk of hepatocellular carcinoma associated with the use of glimepiride compared to individuals who had never taken sulfonylureas. However, there were no significant associations found between exclusive gliclazide use and the occurrence of hepatocellular carcinoma [144]. Gliclazide is reported to possess antioxidant properties and can protect against DNA damage induced by ROS, potentially reducing the risk of cancer [145, 146]. On the other hand, glibenclamide, a potassium ion (K + ATP) channel blocker, may exert anti-tumor effects by blocking K + ATP channels expressed on tumor cells, leading to cell death [147, 148]. The mechanisms governing sulfonylureas' potential anti-tumoral effects versus pro-tumoral effects are not established. Since sulfonylureas constitute a group of compounds, it is crucial to investigate the relationship between each compound and the likelihood of developing cancer.

### Sodium-glucose cotransporter 2 inhibitors

Sodium-glucose cotransporter 2 (SGLT2), a crucial glucose transporter, is often overexpressed in cancer cells, leading to increased glucose uptake in both animal models and humans. Inhibiting SGLT2 expression has proven effective in thwarting in vitro and in vivo tumor development [106]. SGLT2 inhibitors, a relatively new class of antidiabetic drugs, demonstrate anticancer effects in various tumors, including lung, thyroid, pancreatic, breast, and liver cancers [20]. While their primary antidiabetic action is attributed to suppressing glucose uptake, SGLT2 inhibitors exhibit multiple mechanisms to combat cancer, such as down-regulating oxidative phosphorylation, increasing cell cycle arrest and apoptosis, suppressing β-catenin and PI3K-Akt pathways, and causing mitochondrial membrane instability. Additionally, SGLT2 inhibitors reduce tumor invasion and metastasis, inhibit tumor development and proliferation, and enhance the effectiveness of chemotherapy and radiotherapy [20, 106, 149] (Table 2).

An emerging body of evidence and ongoing clinical trials suggest a potential benefit in combining an SGLT2 inhibitor with standard chemotherapy as part of a combination treatment approach. However, additional experimental and clinical data are needed to describe the expression and function of SGLTs in various types of cancer, the activity of various SGLT subtypes, and their role in the growth and progression of tumors [106]. There is a lack of long-term data from human research evaluating the effects of SGLT2 inhibitors on cancer. Suissa et al. conducted a population-based cohort study using the U.K. Clinical Practice Research Datalink (CPRD) and observed no difference in breast cancer incidence between new users of SGLT2 and DPP-4 inhibitors, with a median follow-up of just 2.6 years, suggesting that SGLT2 inhibitors might not have any positive effects on the metabolic changes they are associated with [150]. On the other hand, a meta-analysis of randomized clinical trials revealed a clear association between SGLT2 inhibitors and a notable reduction in the overall risk of cancer compared to a placebo, particularly highlighting the effectiveness of dapagliflozin and ertugliflozin [109].

Evidence from the European Pharmacovigilance Database has raised concerns about an increased risk of bladder cancer in real-world settings among SGLT2 inhibitor users [151]. However, Garcia also suggested ambivalence in the data [151], SGLT2 inhibitors appear to exert their desirable effects by rapidly reducing angiogenesis and environmental survivability, blocking glucose reabsorption, and encouraging urine glucose excretion, which depletes energy supplies for cancer cells [20]. Notably, the anticancer properties of these drugs extend beyond their glucose-lowering benefits. Exploring the additive or synergistic potential of SGLT2 inhibitors in oncology, either as a pretreatment or in combination with established chemotherapeutics, represents a feasible alternative. Ongoing clinical trials are investigating the pleiotropic effects of SGLT2 inhibitors, and delving into their multifaceted impacts opens avenues for new research opportunities. This exploration offers valuable insights into the development of cancer treatments focused on diminishing tumor growth and improving diabetic and cardiac health, ultimately leading to enhanced patient outcomes [20, 106, 149].

### Dipeptidyl peptidase-4 inhibitors

Dipeptidyl peptidase 4 (DPP-4) inhibitors, colloquially known as gliptins, are oral medications designed to lower blood glucose levels in individuals with T2D. These agents block the activity of the DPP-4 enzyme, leading to increased levels of incretins. This dual action suppresses the release of glucagon and prompts the secretion of insulin, ultimately resulting in reduced circulating glucose levels [152, 153]. Beyond their established efficacy in addressing T2D, DPP-4 inhibitors have attracted attention from researchers exploring their potential role in cancer biology [35, 152].

DPP-4 inhibitors, such as vildagliptin, sitagliptin, and saxagliptin, play a crucial role in preserving the integrity of glucagon-like peptide-1 (GLP-1), a hormone vital for regulating glucose levels. In addition to their established function in diabetes management, these inhibitors have demonstrated intriguing potential in cancer treatment [35, 152]. For example, sitagliptin treatment inhibits DPP-4, impeding the transformation of mammary epithelial cells mediated by epidermal growth factor (EGF). This inhibition is linked to a reduction in the expression of PIN1 [154]. Sitagliptin has further shown the ability to stimulate the expression of p21 and p27 while suppressing the activation of PCNA in MCF7 breast cancer cells. Both sitagliptin and vildagliptin exhibit anticancer activity in colon cancer in vitro [155].

Sitagliptin demonstrates the potential to reduce colon cancer risk and blood ROS levels in rats, offering protection against DENA-induced HCC by suppressing inflammation and activating NF-κB [156]. Retrospective studies suggest that after one year of use, sitagliptin may reduce breast cancer risk in T2DM patients and could decrease prostate and oral cancer risk, with effects potentially influenced by dosage and treatment duration [157–159]. In a study by Bishnoi and colleagues, diabetic patients with colorectal and lung cancers who received DPP-4 inhibitors experienced a statistically significant survival benefit (hazard ratio of 0.89; CI: 0.82–0.97, p = 0.007), even after controlling for all confounding factors. Notably, when DPP-4 inhibitors were combined with metformin, the survival advantage was even more prominent (hazard ratio of 0.83; CI: 0.77–0.90, p < 0.0001) [152].

A meta-analysis of 72 randomized controlled trials (RCTs) has shown that individuals with T2DM treated with DPP-4 inhibitors face a significantly reduced risk of developing cancer compared to those receiving a placebo or other chemotherapeutic agents [160]. Vildagliptin, in particular, has demonstrated its ability to inhibit lung cancer growth by enhancing the activity of natural killer (NK) cells mediated by macrophages. This impact is delivered through surfactant-activated macrophages and NK cells, which combat the tumor by employing TRAIL-mediated cytotoxicity [161]. DPP-4 inhibitors have shown anticancer properties in various studies, including their potential to inhibit tumor growth and improve survival rates in cancer patients. While initial concerns about pancreatic cancer risk have been largely addressed, the benefits of DPP-4 inhibitors in both cancer treatment and prevention are increasingly recognized (Table 2).

### Glucagon-like peptide-1 receptor agonists (GLP-1RAs)

GLP-1 (glucagon-like peptide-1) serves as an incretin secretory molecule, regulating insulin secretion. GLP-1 receptor agonists (GLP-1RAs) are commonly used in treating T2DM by enhancing glucose-induced insulin secretion and suppressing hunger [118]. GLP-1 receptors are widespread in pancreatic and other tissues, including various cancer cell types such as those in thyroid, pancreatic, and prostate cancers. The connection between GLP-1RAs and T2DM-related tumorigenesis has garnered attention. While the precise impact of GLP-1 on cancer cells is uncertain, it seems to impede prostate cancer growth. Combining GLP-1 action with metformin treatment appears to have an added beneficial effect in managing prostate cancer [118, 162]. Previous findings suggest that GLP-1 mimetics may possess anti-cancer properties, though the underlying mechanisms need further investigation. For example, Exendin-4 has demonstrated the ability to inhibit the proliferation of human prostate cancer cells by suppressing the ERK-MAPK pathway [163]. In prostate cancer patients, Exendin-4 has been observed to enhance the response to chemotherapy and reduce cancer growth by activating the PI3K/Akt/mTOR pathways [164].

A comprehensive meta-analysis of 37 randomized controlled trials (RCTs) found that albiglutide treatment was associated with a reduced risk of overall cancer (OR 0.76 [95% CI 0.60–0.97]; p = 0.03) [165]. Another meta-analysis of 43 randomized controlled trials demonstrated that GLP-1 receptor agonists (GLP-1 RAs) were not linked to pancreatic cancer (MH-OR 1.28 [0.87, 1.89]; p = 0.20) or pancreatitis [Mantel–Haenszel Odds Ratio (MH-OR) 1.24 [0.94, 1.64]; p = 0.13] [166]. However, a nested case–control analysis using the French National Health Care Insurance System (SNDS) database indicated an elevated risk of all thyroid cancer and medullary thyroid cancer associated with the use of GLP-1 RAs, particularly after 1–3 years of treatment [167]. Larger and long-term studies may be necessary to clarify the effects of GLP-1 RAs and address concerns about the potential impact of study duration on outcomes.

### Thiazolidinediones

Thiazolidinediones (TZDs), a class of antidiabetic drugs, have shown promise in the treatment of breast, thyroid, lung, and prostate cancer through preclinical and clinical studies [14, 168]. This class includes compounds like troglitazone, rosiglitazone, and pioglitazone, with derivatives such as efatutazone and netoglitazone also exhibiting antitumor effects [169]. However, inconsistent findings exist across different studies regarding the antiproliferative effects of TZDs [21, 170–172]. TZDs exert their anti-cancer activity through two pathways: PPAR-γ-dependent and PPAR-γ-independent modes. Activation of the PPAR-γ receptor leads to downstream effects such as reduced proliferation, increased apoptosis, adipocyte differentiation, and elevated adiponectin levels [14, 168, 173]. While TZDs show anticancer potential in certain cancers through these pathways, reports indicate a dual role of PPAR-γ, with evidence of tumor-promoting activation in specific cancers. Independent of PPAR-γ, TZDs also operate by influencing the expression of PTEN/AMPK, AKT/mTOR, and the degradation of cyclins D1 and D3 [14, 173, 174]. Moreover, these drugs function by suppressing the expression of specific target genes, including the insulin receptor gene, prostaglandin E2 receptor gene, and vascular endothelial growth factor gene [175]. Ciglitazone, in particular, is known for its ability to decrease aromatase activity in androgen-dependent prostate cancer [176, 177].

### α-Glucosidase inhibitors

α-Glucosidase is considered a prime drug target for diabetes and its inhibitors are used to delay carbohydrate digestion for the treatment of diabetes. It slows down the digestion of complex carbohydrates by blocking the α-glucosidase enzyme located at the brush border of the small intestines. They are mainly used for the reduction of postprandial hyperglycemia by suppressing glucose absorption [178] (Table 2). Besides, the current interest in α-Glucosidase inhibitors has been extended to a broad range of diseases including cancer [179]. In a nationwide population-based study involving diabetic patients with colorectal cancer, the use of acarbose, an α-glucosidase inhibitor was associated with a significant 27% reduction in cancer risk. The risk reduction was dose-dependent, with adjusted hazard ratios of 0.73 (95% CI 0.63–0.83), 0.69 (0.59–0.82), and 0.46 (0.37–0.58) for increasing cumulative doses of acarbose compared to non-users (p for trend < 0.001) [180].

A meta-analysis of observational studies has suggested that the use of α-glucosidase inhibitors is associated with a reduced risk of cancer, with an odds ratio (OR) of 0.86 and a 95% confidence interval (CI) of 0.78–0.96. This association appears to be more pronounced in gastrointestinal cancer, with an OR of 0.83 and a 95% CI of 0.71–0.97. However, the results across studies showed variability, which can be attributed to differences in study quality and the extent of adjustment for potential confounding factors. It's noteworthy that a meta-analysis of randomized controlled trials did not find a significant association between α-glucosidase inhibitors and cancer risk [126]. While existing studies suggest a potential protective association between the use of α-glucosidase inhibitors and cancer risk in individuals with diabetes, further well-designed prospective research is needed to confirm and better understand this association.

## Do anti﻿diabetic medications have a similar line of action in non-diabetic cancer patients?

The investigation into the potential use of antidiabetic drugs in cancer treatment has prompted a critical question: do these medications exhibit comparable modes of action in non-diabetic cancer patients? Although traditionally prescribed for diabetes management, emerging evidence suggests that certain antidiabetic drugs may hold therapeutic potential in cancer treatment. Metformin has been extensively studied for its anti-cancer properties. Multiple studies indicate a tendency toward reduced incidence of various cancers. Enhanced outcomes have been observed in non-diabetic cancer patients with specific histological subtypes or genotypes undergoing treatment with metformin either alone or in combination with other therapies. However, available results from prospective and randomized trials are limited. Additionally, investigations into the immunomodulatory properties of metformin on cancer cells should be considered to optimize its clinical utilization [181]. A recently published randomized controlled study has revealed that metformin provides a therapeutic avenue for managing toxicities resulting from neoadjuvant chemotherapy in breast cancer patients without diabetes [182]. In preclinical studies, metformin's growth inhibitory effects primarily stem from proliferation inhibition rather than inducing extensive apoptosis or necrosis. This implies that metformin alone may not achieve complete tumor remission, leading to an increasing focus on combining it with other therapies. Studies across various cancer types combining metformin with standard treatments or other drugs have shown superior responses compared to metformin monotherapy. However, understanding the mechanisms of synergy between metformin and other anti-cancer agents remains unclear, necessitating further exploration in prospective clinical trials [86, 183–185].

Other antidiabetic medications, including sulfonylureas, SGLT2 inhibitors, DPP-4 inhibitors, GLP-1RAs, TZDs, and α-glucosidase inhibitors, have shown compelling effects in preclinical studies. However, it is important to note that the primary body of evidence originates from diabetic populations. Thus, a thoughtful approach is essential when transitioning these findings into clinical practice, despite promising preclinical results. Further research is indispensable to determine the applicability of these insights to prediabetic or non-diabetic individuals. It is imperative to identify specific populations that would yield the most favorable benefit-to-risk ratio, ensuring the safe and effective application of antidiabetic drugs in the broader context of cancer treatment [78]. Another crucial consideration lies in optimizing doses for non-diabetic patients, a factor that can be pivotal. Employing lower doses than those conventionally used in diabetic treatment could enhance effectiveness while minimizing side effects. Additionally, the timing of administering these drugs alongside standard cancer therapy appears to be influential, offering another avenue for potential optimization.

## Challenges and limitations

Diabetes is a chronic metabolic disorder whereas cancer is a highly heterogeneous and genetic disease. They share multiple common risk factors and their manifestations are observed in multiple organs [186]. The complexity of cancer demands a multi-dimensional treatment strategy. Hence, repurposed use of known old drugs appears to be useful. Also, epidemiological studies suggest that diabetic patients are prone to develop some specific types of cancers as mentioned in the introduction Sect.  [187]. It raises a question on the role of antidiabetic drugs in combating cancer for diabetic patients who were already consuming antidiabetic drugs and developed cancer. Overall, effects of each type of antidiabetic drugs in cancer initiation, promotion and progression needs more better understanding [188]. Additionally, diabetes may develop in cancer patients due to the cancer or therapies used for cancer [189]. Pancreatic cancer often causes diabetes, considered as type 3c diabetes when pancreas fails to produce sufficient insulin [190, 191]. Cancer patients are often treated with steroids during chemotherapy which may cause secondary diabetes worsening the quality of life of the patients [192–194]. The role of antidiabetic drugs in combating both diseases in such scenarios needs to be explored. Moreover, the biological relationships of the two diseases are still not fully understood [11]. It makes the clinical management challenging for patients suffering both cancer and diabetes [195]. It sets further questioning to use antidiabetic drugs as repurposed drugs for cancer. The heterogeneity and personalized nature of cancer make this challenge crucial.

To date, several clinical trials have been conducted targeting the repurposed use of antidiabetic drugs in the case of cancer. It is also observed that their outcomes are often diverging. The response of antidiabetic drugs depends on whether the cancer patient is diabetic or not [196]. In the case of metformin, several studies and clinical trials have already reported promising results. However, for some clinical trials metformin failed to provide any benefit compared to the standard chemotherapy [197]. The specific molecular mechanism behind the anti-cancer role of metformin is still unknown. Some studies have also miss-interpreted the trial outcomes [99]. Also, the appropriate dose and administration time of metformin need to be enquired. In the case of sulfonylureas, depending on the type of sulfonylureas the risk of cancer increases [144]. Certain inhibitors of SGLT2 appear as a promising anti-cancer agent. However, its molecular anti-cancer mechanism and clinical feasibility require more pre-clinical as well as clinical studies for different types of inhibitors and cancers [106, 198]. Ng et al. reported that the colorectal cancer patients treated with DPP-4 inhibitors showed better 5-year prognosis compared to the patients treated with metformin [199]. Hence, the selection of proper antidiabetic drugs needs to be addressed better. TZDs have also reported conflicting results [121]. The conflicting results are also observed in the case of AGIs [126]. Several inconsistent reports set the limitation of the repurposed use of antidiabetic drugs. As these drugs are supposed to be used as adjuvants with other therapeutics including chemotherapy, radiotherapy, and immunotherapy, the appropriate combinative therapy needs to be defined [19]. The implications of antidiabetic drugs with cancer immunotherapy needs to be explored. It is observed that effects of antidiabetic drugs on anti-PD1 immune checkpoint inhibitors vary widely [200, 201]. The combination of several antidiabetic drugs is also being explored to treat cancer. Yang et al. reported the possibility of adverse effects when the combination of GLP-1RA and DPP-4 inhibitors is used [202]. The site of cancer, stage of the disease, and mutational profile are also determinant factors on the effectiveness of repurposed use of antidiabetic drugs. The patient specific mutational landscapes are also crucial. High glucose levels enhances mutation rate and decreases DNA damage repair efficiency by affecting DNA damage response pathway in the case of breast cancer [203–205]. Occurrence of cancer among diabetes patients is more prone among the younger age group (40 – 54 years) [206]. Hence, defining appropriate dose and duration is also a challenge. It requires a personalized approach of treatment. Among different cancer types, breast, lung, colon, and prostate are the most common cancer sites and studied [207]. The association of diabetes with rare cancers also need to be studied. In the context of preventive measures, prevention of diabetes will definitely reduce cancer occurrence [208].

## Conclusion

The increasing occurrence of cancer is a concern to healthcare management across the globe. The concern is not only for the loss of lives but also for the affordability of the treatment cost. The complexity of the disease itself requires a variety of drugs. The increasing price, and availability of cancer drugs are limitations in maintaining a minimalist quality of life for cancer patients. In this context, several known drugs are repurposed to provide benefits to cancer patients at a reduced cost of money and time. Antidiabetic drugs regulating metabolic mechanisms are considered first-line choices for repurposed use in treating cancer as both diseases have wide overlap in the underlying biological pathways.

Repurposing antidiabetic drugs for cancer treatment holds great potential, driven by their well-established safety profiles, expedited development timelines, potential cost-effectiveness, and targeting pathways such as PI3K/AKT/mTOR, Wnt/β-catenin, JAK/STAT in cancer, and AMPK and PPARγ in diabetes to accelerate clinical application. Metformin stands out as a frontrunner, demonstrating anti-proliferative, pro-apoptotic, and chemo-sensitizing properties. However, the landscape varies across antidiabetic classes. Sulfonylureas and TZDs lack consistent anti-cancer activity, while SGLT2 and DPP-4 inhibitors exhibit preliminary promise. The potential cancer risk associated with GLP-1RAs necessitates further investigation. The existing evidence regarding variations in cancer risk among antidiabetic medications lacks the robustness required to direct clinical decision-making. Consequently, future research should delve deeper into the mechanisms underlying the anti-cancer effects of different antidiabetic drugs and conduct robust clinical trials to establish the efficacy and safety of repurposed antidiabetics in specific cancer contexts.

The occurrence of diabetes is also increasing at an alarming rate. Diabetes also increases the risk of certain types of cancers and induces different co-morbidities. Hence, effectiveness of the antidiabetic drugs in treating cancer, depends on the co-morbidities, patient’s conditions, stages of the co-morbid diseases, and on the conjunction of other drugs. The role of antidiabetic drugs within tumor microenvironment needs more studies. Involvement of such diverse factors produces contradictory results of using any drug in different patients. Hence, the successful repurposed use of drugs requires disease management with a comprehensive personalized approach.

## Data Availability

Not applicable.
